# Visual stimulus parameters seriously compromise the measurement of approximate number system acuity and comparative effects between adults and children

**DOI:** 10.3389/fpsyg.2013.00444

**Published:** 2013-07-19

**Authors:** Dénes Szűcs, Alison Nobes, Amy Devine, Florence C. Gabriel, Titia Gebuis

**Affiliations:** ^1^Department of Psychology, Centre for Neuroscience in Education, University of CambridgeCambridge, UK; ^2^Department of Experimental Psychology, Katholieke Universiteit, KU LeuvenLeuven, Belgium

**Keywords:** mathematical development, number sense, magnitude comparison, ANS, numerical cognition, Weber fraction, dyscalculia

## Abstract

It has been suggested that a simple non-symbolic magnitude comparison task is sufficient to measure the acuity of a putative Approximate Number System (ANS). A proposed measure of the ANS, the so-called “internal Weber fraction” (*w*), would provide a clear measure of ANS acuity. However, ANS studies have never presented adequate evidence that visual stimulus parameters did not compromise measurements of *w* to such extent that *w* is actually driven by visual instead of numerical processes. We therefore investigated this question by testing non-symbolic magnitude discrimination in seven-year-old children and adults. We manipulated/controlled visual parameters in a more stringent manner than usual. As a consequence of these controls, in some trials numerical cues correlated positively with number while in others they correlated negatively with number. This congruency effect strongly correlated with *w*, which means that congruency effects were probably driving effects in *w*. Consequently, in both adults and children congruency had a major impact on the fit of the model underlying the computation of *w*. Furthermore, children showed larger congruency effects than adults. This suggests that ANS tasks are seriously compromised by the visual stimulus parameters, which cannot be controlled. Hence, they are not pure measures of the ANS and some putative *w* or ratio effect differences between children and adults in previous ANS studies may be due to the differential influence of the visual stimulus parameters in children and adults. In addition, because the resolution of congruency effects relies on inhibitory (interference suppression) function, some previous ANS findings were probably influenced by the developmental state of inhibitory processes especially when comparing children with developmental dyscalculia and typically developing children.

It has been suggested that a non-symbolic magnitude comparison task is sufficient to measure the acuity of a putative Approximate Number System (ANS). A proposed measure of the ANS, the so-called “internal Weber fraction” (*w*), should provide a clear measure of ANS acuity. However, ANS studies relying on *w* as a sole measure of the acuity of the ANS (Piazza et al., 2004, 2010; Halberda and Feigenson, 2008; Halberda et al., 2008; Mazzocco et al., 2011) have never presented adequate evidence that visual stimulus properties (e.g., surface, density) do not seriously compromise measurements and have taken it for granted that experimental controls for non-numerical parameters were adequate. However, this has been shown to be an invalid assumption and in fact, non-numerical parameters cannot be controlled in each individual trial (Gebuis and Reynvoet, 2012a,b). In order to examine the influence of the visual stimulus properties on performance, that is, to determine the validity of ANS measures, we used a non-symbolic magnitude discrimination paradigm, which used even more stringent controls of visual parameters than usual. Next we investigated the effect of these visual manipulations by comparing the trials where the visual stimulus properties correlated either positively or negatively with numerical parameters and examined the impact of this manipulation on *w*. Further, we examined how the impact of visual confounds on *w* differs between adults and children.

Several researchers have assumed that we are equipped with an ANS that allows us to compare or judge the numerosity of different sets of items independent of the visual properties of these items (e.g., Halberda and Feigenson, 2008; Piazza et al., 2010). Studies aimed to determine the precision of the ANS by giving participants a simple non-symbolic magnitude discrimination task and computing *w* which represents the standard deviation (logarithmic models) or a factor in the standard deviation (linear models) of Gaussian tuning curves for the representation of numerosities (Piazza et al., 2004). Piazza et al. (2010) define *w* as: “… the “internal Weber fraction” … [which] measures the precision of the internal representation and is therefore a sensitive index of number acuity” (p. 34). Or, Mazzocco et al. (2011) describe *w* as: “The amount of noise in an individual's Approximate Number System is indexed as a Weber fraction (*w*). This index can be derived by asking the individual to evaluate which of two quickly flashed arrays of objects is more numerous…” (p. 2).

In non-symbolic magnitude discrimination tasks participants are typically asked to compare two numerosities (the number of presented items) and press a button on the side where they see more items. *w* is then computed by fitting a sigmoid function describing discrimination performance (the percent of “larger” responses in the task). Obviously, when the participant presses a button on the side where there are indeed more items, the “larger” response is correct. In contrast, when the participant presses the button on the side where there are in fact less items, the “larger” response is incorrect. Hence, decision curves exactly equal accuracy (percent correct) when the ratio of the to-be-compared numerosity to the reference numerosity is larger than one (because a >1 ratio means that the to-be-compared numerosity is indeed larger than the reference number; e.g., 18 compared to a reference of 12: 18/12 = 1.5). In contrast decision curves equal 1 minus accuracy in the part of the curves where ratios are smaller than 1 (because a <1 ratio means that the to-be-compared numerosity is in fact smaller than the reference number; e.g., 9 compared to a reference of 12: 9/12 = 0.75). *w* provides a measure of the (sigmoid) shape of the decision curves. It is important to realize that *w* is a direct function of accuracy data recorded in an experiment. Figure 1 depicts example decision curves and related accuracy curves. The sigmoid function fitted to the data can be based on assuming either a linear or a logarithmic number line. In typical human experiments both assumptions lead to similar results. Hence, we used the linear number line version of the equations as this is used more frequently in developmental research. The function is described by e.g., Halberda et al. (2008) as:

$$\text{Proportion Judged Larger }(n1,n2)=\frac{1}{2}\cdot \text{erfc }\left(\frac{n2-n1}{\sqrt{2}\;w\;\sqrt{n1^{2}+n2^{2}}}\right)$$

where *n*1 is a numerosity compared to *n*2, the reference numerosity and erfc is the complementary error function, a well-known mathematical function.

**Figure 1:**
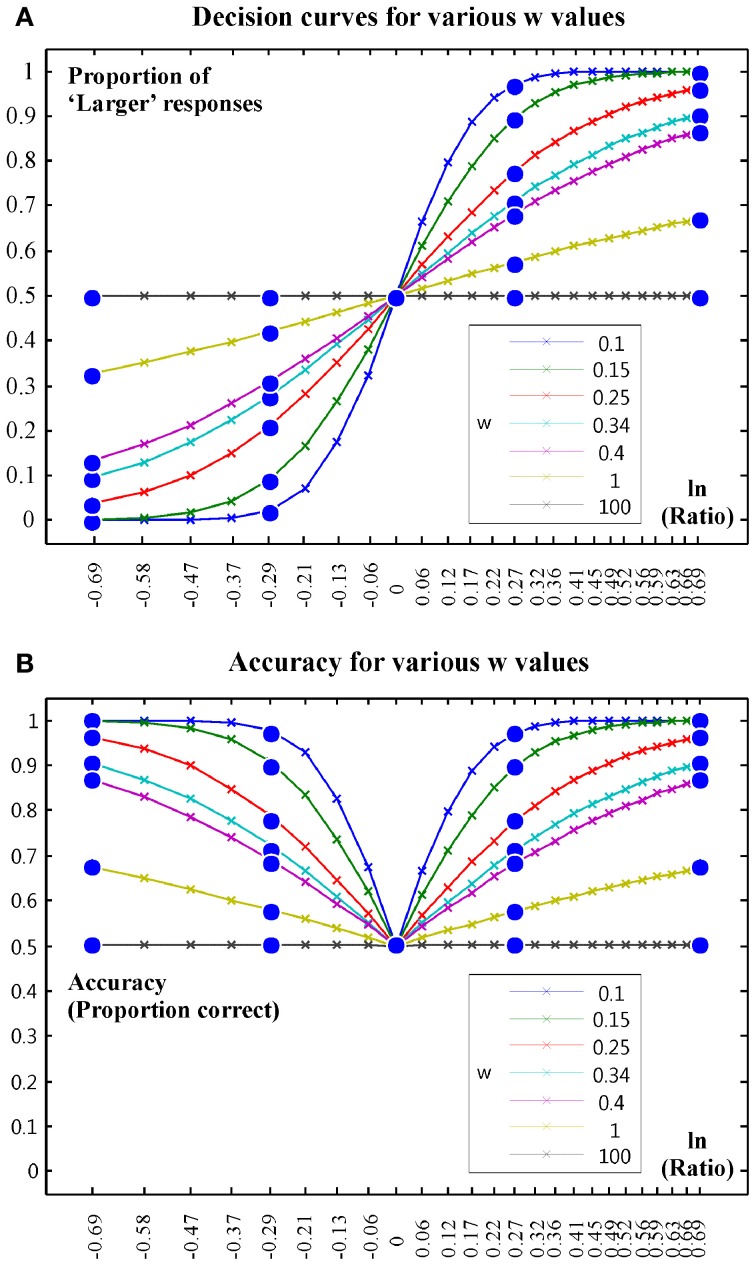
**Illustration of decision curves and accuracy outcomes for various *w*-values. (A)** Decision curves for various w values. **(B)** Accuracy for various w values.

A common problem with ANS studies relying on *w* (Piazza et al., 2004, 2010; Halberda and Feigenson, 2008; Halberda et al., 2008; Mazzocco et al., 2011; Halberda et al., 2012) is that they have never considered the possibility that controls over visual stimulus parameters might not be sufficient. The general method to control the visual stimulus parameters in non-symbolic number comparison tasks is to create the stimuli in such a manner that each single visual parameter is not informative about number across all trials. To achieve this goal, researchers created stimuli where the visual stimulus properties were larger for the larger number in half of the trials (congruent trials) and smaller for the larger number in the other half of the trials (incongruent trials). There are two major problems with these designs. First, applying these visual controls only accounts for reliance on a *single* visual cue across all trials. It does not eliminate the possibility that participants are influenced by or rely on different visual cues in every single trial. Nor does it account for the possibility that participants integrate multiple visual stimulus properties at the same time. Recently, it was shown that the visual congruency effect in a numerosity task increases when more visual parameters are present in the stimuli, implicating that participants integrate multiple visual cues to perform a numerical taks (Gebuis and Gevers, 2011; Gebuis and Reynvoet, 2012a). Second, these manipulations of the visual stimulus properties do not control for the relation between the difference in visual properties and the difference in number. Often the difference in visual properties increases with increasing distance in numerosity. Hence, the presence of a ratio effect, which is always held as evidence for numerical processes, does not necessarily imply that number caused these results. Further, as the visual stimulus properties cannot be controlled in an individual trial, there are always visual cues in a display that correlate with number (i.e., there is no truly “neutral” condition where visual cues do not correlate with number in any particular trial), it is therefore necessary to evaluate the effect of the visual controls applied.

In order to gain an explicit impression about the effect that visual stimulus parameters have on performance in non-symbolic number comparison tasks, one could look at the congruency effect, which is the difference in performance between congruent and incongruent trials. In the congruent trials numerical and visual information provide similar information, that is, numerical and visual cues are positively correlated. In contrast, in incongruent trials numerical and visual information provide opposing information, that is, numerical and visual cues are negatively correlated. It is important to realize that a non-symbolic discrimination task where in some trials visual parameters correlate positively with number and in other trials visual parameters correlate negatively with number in effect becomes a Stroop task. Participants basically have to discriminate along a task relevant numerical dimension while they are supposed to neglect task-irrelevant non-numerical (visual) parameters of displays. In fact, because it is impossible to control every particular trial for visual parameters, each trial in each non-symbolic numerosity discrimination task can be considered a Stroop task trial. In general, worse performance can be expected in the incongruent condition than in the congruent condition (e.g., Mix et al., 1997; Rousselle et al., 2004; Soltész et al., 2010). However, a number of studies also showed the opposite pattern: better performance in incongruent than congruent trials (Miller and Baker, 1968; Ginsburg and Nicholls, 1988; Sophian, 2007; Gebuis and Reynvoet, 2012a; Gebuis and van der Smagt, 2011). This suggests that the association between numerical and physical size is not rigid and might depend on the method used to control the visual stimulus parameters.

Congruency effects can be explained in various, not necessarily mutually exclusive, ways. A sensory explanation could be that people may be unable to extract numerical information correctly from visual displays but instead rely on the visual stimulus parameters to judge numerosity. In this case, chance, or below chance performance in the incongruent condition could be interpreted to demonstrate weak ANS function or no ANS function at all. Below chance performance in the incongruent condition would suggest that people carried out the task primarily relying on visual cues rather than on number. Another, executive function related, explanation of congruency effects would be that people may get distracted by task-irrelevant visual information in the incongruent condition because they cannot focus their attention properly on task-relevant parameters, or because they cannot inhibit incorrect response tendencies efficiently and may ultimately press the wrong response button. In this case, a larger congruency effect in children than in adults would be interpreted to demonstrate some worse executive function (e.g., attention or inhibition) in children than in adults. In fact, congruency effects in Stroop-like tasks in children are frequently used as measures of inhibition function (Gerstadt et al., 1994; Huizinga et al., 2006). A general finding is that children show larger congruency effects (congruent vs. incongruent difference) than adults with practically any kind of stimulus material. This has usually been attributed to worse attentional focusing or worse inhibition function in children than in adults (Gerstadt et al., 1994; Bunge et al., 2002; Szűcs et al., 2007, 2009; Bryce et al., 2011).

Independently from the exact explanation of congruency effects, larger congruency effects in children than in adults can be expected in non-symbolic comparison data: children can be expected to have lower accuracy scores in incongruent trials than adults. Consequently, if a single accuracy score (the mean score from congruent and incongruent trials) is computed then children's worse performance in incongruent trials will manifest itself in a lower total accuracy score than in adults. This lower accuracy score then will be fed into an algorithm producing *w* and will result in a *w* score which is higher for children than for adults (smaller accuracy scores result in larger *w*-values and vice versa). This larger *w*-value in children can then be interpreted as an expression of a less accurate ANS when in fact it is a consequence of a larger congruency effect in children than in adults. As noted above, such a larger congruency effect is the consequence of larger sensitivity to perceptual confounds in children than in adults, or to worse attentional, inhibition or response organization processes in children than in adults. That is, should congruency effects impact the computation of *w, w* should reflect completely non-numerical variables and any potential ANS-related developmental effects would be illusory.

The above potential problem was never investigated properly with regard to *w*. Only one child ANS study using *w* employed a congruency factor (called “stimulus type”) with regard to accuracy (Halberda and Feigenson, 2008). However, the impact of congruency on *w* was not examined. Nevertheless, it was noted that 3, 4, and 5 year-olds had better performance in the congruent than in the incongruent condition. At the same time, these children had worse (larger) *w* than older children. Because *w* is a direct function of accuracy, as the above argument suggests, it is likely that the accuracy difference between congruent and incongruent conditions contributed to *w*-values computed from both congruent and incongruent trials. Further, this study used different stimulus arrangements (patterns presented on the left and right) relative to other studies from the same authors (differently colored dot patterns presented intermixed; Halberda et al., 2008; Mazzocco et al., 2011). Hence, generalizability to other task contexts is not clear.

Here, we compared the magnitude discrimination performance of 7-year-old children and adults. We used a non-symbolic magnitude discrimination task, which employed stringent controls for visual parameters correlated with numerosity. In addition to the general manipulations we also controlled for the correlation between the difference in sensory properties and the difference in numerosity in stimuli across all trials. We used a congruent condition in which certain visual stimulus parameters were positively correlated with number and an incongruent condition in which certain visual stimulus parameters were negatively correlated with number. This allowed us to examine the effect of congruency on accuracy data and on *w* in an explicit manner. First, if participants can discriminate magnitudes independently of visual parameters then we could expect good performance in the incongruent condition of the task. If, on the other hand, participants mostly rely on visual cues when making decisions performance in the incongruent condition may fall well below chance especially for the most difficult ratio conditions. An important question was how well the sigmoid model underlying *w* calculation would fit the data in the congruent and incongruent conditions and when both conditions are collapsed. We expected much worse fits in the incongruent than in the congruent condition. Our second major interest was to study the influence of congruency effects on *w* computed from the data with collapsed congruent and incongruent conditions. We studied this by computing the correlation between the congruency effect and *w*. As discussed above, we expected that congruency effects which are not number specific will have major impact on *w*, a proposed measure of ANS acuity.

## Methods

### Participants

We tested 32 children from Year 2 of primary school and 23 adults in Cambridge, UK. We tested 7-year-olds because they already have a firm number concept (Rubinsten et al., 2002) and they also show more robust inhibitory control than less than 6-year-old children (Gerstadt et al., 1994). Following the practice of Piazza et al. (2010) participants with inferior fit of model-based decision curves to data (*R*^2^ < 0.2) were excluded from analysis. In practice, such a low *R*^2^-value means that accuracy is very low (≤54% in all participants), i.e., exclusion is justified. Nine children were excluded because of low *R*^2^. Two additional children and one adult had accuracy ≤55%. One child did not seem to follow instructions properly scoring <50% in the congruent condition. These participants were also excluded from analysis. Hence, 20 children (mean age: 7 years and 5 months) and 22 adults (25 years and 9 months) were left in the final sample. The excluded and non-excluded children were compared on their Wechsler Achievement Test II (WIAT-II) Numerical Operations scale and on the Raven Colored Progressive Matrices test results. There were no differences between the groups (Means and standard deviations: Included children: WIAT-II: 105 ± 17; Raven CPT: 102 ± 15. Excluded children: WIAT-II: 107 ± 18; Raven CPT: 108 ± 16. Test for WIAT-II: *p* = 0.66; Test for Raven: *p* = 0.33.). Further, it is to note that *w* studies with young children have similarly high exclusion rates (this will be discussed in the Discussion).

### Task and stimuli

We created the stimuli using the program developed by Gebuis and Reynvoet (2011, 2012a). The stimuli consisted of two arrays of gray dots separated by a vertical gray line and were presented on a black background. Two different sets of stimuli were created. We manipulated five different sensory cues to decrease the confound between numerosity and its sensory properties: (1) surface: total surface of the dots in one dot array, (2) diameter: the average diameter of the dots in one dot array, (3) contour length: the total contour length of all dots in one dot array, (4) convex hull: the smallest contour that can be drawn around the dots on one dot array, and (5) density: surface divided by convex hull. In the first set, in half of the trials the different sensory cues comprising the dot patterns (convex hull, diameter, surface, density and contour length) were larger for the larger number and in the remaining half of the trials they were smaller for the larger number. In the second set, in half of the trials some sensory cues were larger for the larger number (density, surface, diameter, and contour length) while others were smaller (convex hull), the reverse was true for the remaining half of the trials. More specifically, surface, diameter and contour length are correlated with each other, when of them changes the others change as well. In contrast, convex hull can be manipulated without changing these visual parameters. This allowed us to create stimuli that were partly congruent and partly incongruent with numerosity. In this manner no single visual cue is informative about number across all trials. In both sets of stimuli we also manipulated the difference between the sensory properties of both arrays in relation to the difference in number. For example, the difference in surface between two sets of stimuli with a large numerical distance (e.g., 12 and 32) is not necessarily larger than the difference in surface between two sets of stimuli with a small numerical distance (e.g., 12 and 18). We created the stimuli in such a manner that across all trials no relationship exists between the difference in sensory properties and the difference in number (For both sets of stimuli all *R*^2^'s < 0.06). Initial analyses revealed no significant differences between responses to the two sets of stimuli. Hence, they were collapsed for further analyses reported here.

One of the two dot arrays contained 16 dots while the other dot array contained a smaller (i.e., 8, 10, 12, 13, or 14 dots) or a larger (i.e., 18, 20, 22, 26, or 32) number of dots. This resulted in 10 different number pairs and 10 different ratios (ratio 0.5, ratio 0.62, ratio 0.74, ratio 0.81 and ratio 0.88; and pairs with 1 divided by the previously listed 5 ratios). In effect, trials with ratios <1 and >1 belonged to 5 levels of Ratio (0.5, 0.62, 0.74, 0.81, 0.88). The trials were presented in randomized order. In half of the trials the larger number of dots was presented on the left side of the screen and in the other half it was presented on the right side. Participants were instructed to indicate which dot array contained more dots by pressing the key corresponding to the side that represented the larger number. Adults had 800 stimuli, i.e., the 10 different number pairs were presented 80 times each (160 trials for each of the 5 ratio conditions). Children had 200 stimuli i.e., the 10 different number pairs were presented 20 times each (40 trials for each of the 5 ratio conditions).

Before the experiment started, participants had 40 practice trials. Each trial started with a fixation cross (1000 ms duration), followed by the stimuli. The stimuli disappeared when a response was given and next the inter-trial interval (500 ms) started. Stimulus presentation and recording of the data were controlled using Presentation 15, Neurobehavioral Systems (www.neurobs.org).

### Fitting model to data

Model decisions curves were generated according to the equations given by Piazza et al. (2010) and Halberda et al. (2008) as described in the introduction:

$$\text{Proportion Judged Larger}(n1,n2)=\frac{1}{2}\cdot \text{erfc }\left(\frac{n2-n1}{\sqrt{2}\;w\;\sqrt{n1^{2}+n2^{2}}}\right)$$

Model decision curves were generated for all *w*-values between 0.01 and 10 in steps of 0.01. A least squares algorithm found the model decision curve best fitting the data of each individual participant and the appropriate *w*-value. The above procedure was run for the whole data with congruency conditions collapsed, as well as for the congruent and incongruent conditions separately.

### Statistics

The <1 and >1 ratio values were collapsed for analysis into 5 levels of Ratio (0.5, 0.62, 0.74, 0.81, 0.88). Accuracy and RT data were analyzed by mixed design Group (Child vs. Adult) × Congruency (congruent vs. incongruent) × Ratio (5 levels) Analysis of Variance (ANOVA). *W*-values were analyzed by a Group × Congruency ANOVA. Greenhouse-Geisser epsilon (ε) correction was used when necessary. Original *F, df* and corrected *p*-values are provided. Effect size is indicated by partial eta-squared values (η^2^_*p*_). The relationship of the congruency effect (congruent minus incongruent values) in accuracy and RT and w; as well as the relationship of total accuracy and the slope of the ratio effect (mean differential of all Ratio levels) were analyzed by Spearman rank correlations (due to the fact that *w* is a non-linear function of accuracy). Linear functions were also fitted to original and log transformed *w* data expressed as a function of the above variables. Analyses were done in Matlab and in Statistica 10.

## Results

**Figure 2:**
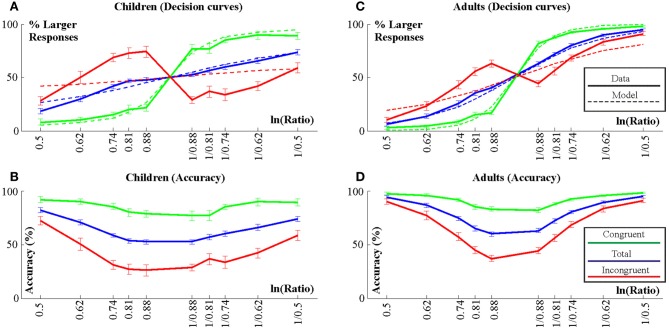
**Decision curves (A and C) and accuracy values (B and D) for children (A,B) and adults (C,D).** Empirical decision curves are shown by thick lines. These curves show standard errors for each ratio level. Model decision curves are shown by thin dashed lines.

**Figure 3:**
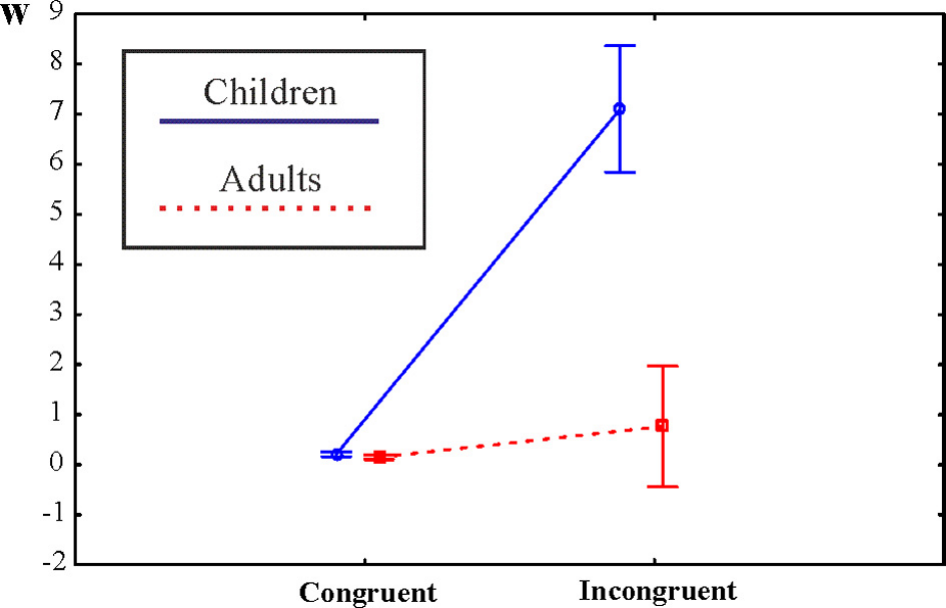
**Illustration of a Group × Congruency ANOVA on *w*-values.** Ninety-five percentage confidence intervals are represented.

As noted, overall accuracy was >55% in each individual. Empirical and model decision curves are shown in Figures 2A–C. Accuracy is shown in Figures 2B–D. As noted, empirical decision curves and accuracy represent exactly the same data in different ways. Mean *w*-values and ranges of *w*-values for children and adults are shown in Table 1. The first ANOVA with a Group factor was run on *w*-values computed from overall accuracy. Children differed from adults [*F*_(1, 40)_ = 42.7; η^2^_*p*_ = 0.52; *p* < 0.001; children: *w* = 0.77 ± 0.05; adults: *w* = 0.30 ± 0.05). Figure 3 illustrates the outcomes of the Group × Congruency ANOVA. Children differed from adults [*F*_(1, 40)_ = 56.39; η^2^_*p*_ = 0.59; *p* < 0.001] and there was a Congruency effect [*F*_(1, 40)_ = 73.3; η^2^_*p*_ = 0.65; *p* < 0.001] and a Group × Congruency interaction [*F*_(1, 40)_ = 58.3; η^2^_*p*_ = 0.56; *p* < 0.001]. Group × Congruency Tukey *post-hoc* contrasts demonstrated that the interaction appeared because children and adults did not differ in the congruent condition (*p* = 0.99; children: *w* = 0.18 ± 0.02; adults: *w* = 0.13 ± 0.02) but children had much more positive *w*-values than adults in the incongruent condition (*p* < 0.001; *w* = 7.10 ± 0.62; adults: *w* = 0.74 ± 0.60). The congruency effect was significant in both groups (*p* < 0.001).

**Table 1:** ***W*-values in children and adults**.

	**Children**					**Adults**				
	**Mean**	**Minimum**	**Maximum**	**Conf Int −95%**	**Conf Int +95%**	**Mean**	**Minimum**	**Maximum**	**Conf Int −95%**	**Conf Int +95%**
**Total**	0.77	0.39	1.59	0.62	0.93	0.30	0.20	0.52	0.26	0.34
**Congruent**	0.19	0.01	0.57	0.12	0.26	0.13	0.06	0.28	0.11	0.15
**Incongruent**	7.11	0.46	10.00	5.23	8.98	0.74	0.25	2.75	0.42	1.06

**Figure 4:**
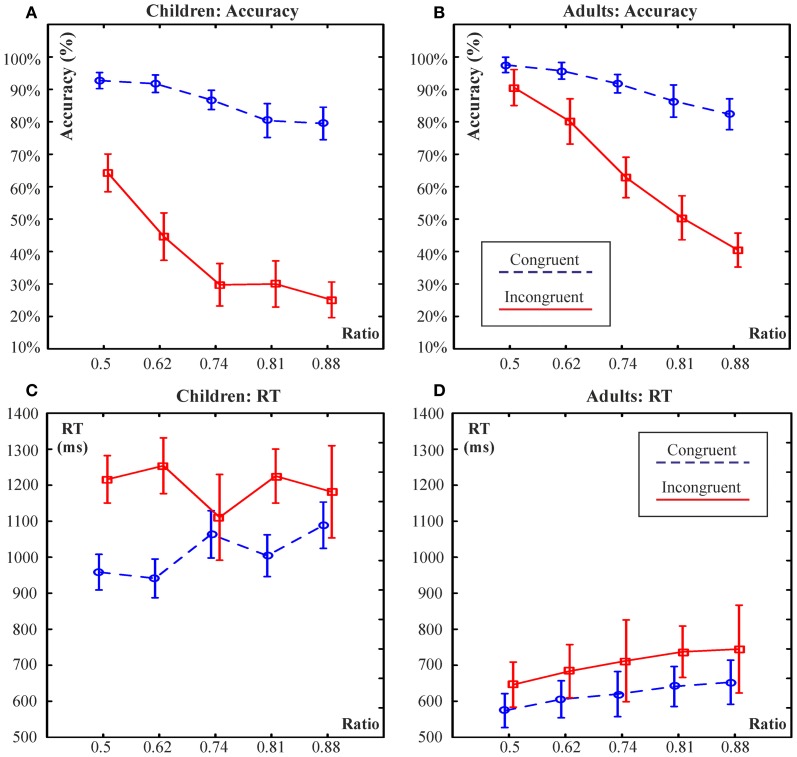
**Illustration of a Group × Congruency × Ratio ANOVA on accuracy (A,B) and reaction time (C,D) data.** Ninety-five percentage confidence intervals are represented.

Accuracy data was evaluated by a Group × Congruency × Ratio ANOVA. Results are illustrated in Figures 4A,B. All ANOVA effects were significant. Children were less accurate than adults [62.5 ± 1% vs. 77.8 ± 1%; Group: *F*_(1, 40)_ = 89.3; η^2^_*p*_ = 0.69; *p* < 0.001]. There was a strong effect of Congruency [*F*_(1, 40)_ = 193.6; η^2^_*p*_ = 0.83; *p* < 0.001] and a Congruency × Group interaction [*F*_(1, 40)_ = 16.7; η^2^_*p*_ = 0.30; *p* < 0.001]. The interaction was the result of a larger congruency effect in children than in adults: According to Congruency × Group Tukey *post-hoc* contrasts, both groups showed a congruency effect (Congruent vs. incongruent difference in both: *p* < 0.001). Accuracy did not differ by group in the congruent condition (*p* = 0.46; mean and SE: children: 86.2 ± 4%; adults: 90.8 ± 4%) whereas it was lower in children than in adults in the incongruent condition (*p* = 0.0001; 38.7 ± 3% vs. 64.8 ± 6%).

There was a Ratio effect [*F*_(4, 160)_ = 254.1; ε = 0.854; η^2^_*p*_ = 0.86; *p* < 0.001] and there were Group × Ratio [*F*_(4, 160)_ = 8.5; ε = 0.854; η^2^_*p*_ = 0.18; *p* < 0.001], Congruency × Ratio [*F*_(4, 160)_ = 62.2; ε = 0.725; η^2^_*p*_ = 0.61; *p* < 0.001] and Group × Congruency × Ratio [*F*_(4, 160)_ = 7.13; ε = 0.725; η^2^_*p*_ = 0.15; *p* < 0.001] interactions. Group × Congruency × Ratio Tukey contrasts showed that in the congruent condition none of the Ratio levels differed between children and adults (*p* > 0.88). However, in the incongruent condition all Ratio levels were responded less accurately by children than by adults (*p* < 0.001). Further, in children in the incongruent condition accuracy did not differ at the 3 difficult ratios (0.74, 0.81, 0.88). Comparing 0.74 vs. 0.81 and 0.81 vs. 0.88: *p* > 0.81. Accuracy differed between the 3 least difficult ratio levels. Comparing 0.5 vs. 0.62 and 0.62 vs. 0.74: *p* < 0.001. In contrast, in adults all ratio levels differed from each other: *p* < 0.001 for all comparisons. The above means that children's performance dropped to a lower level than that of adults at the 3 most difficult ratio levels, which did not differ from each other.

**Figure 5:**
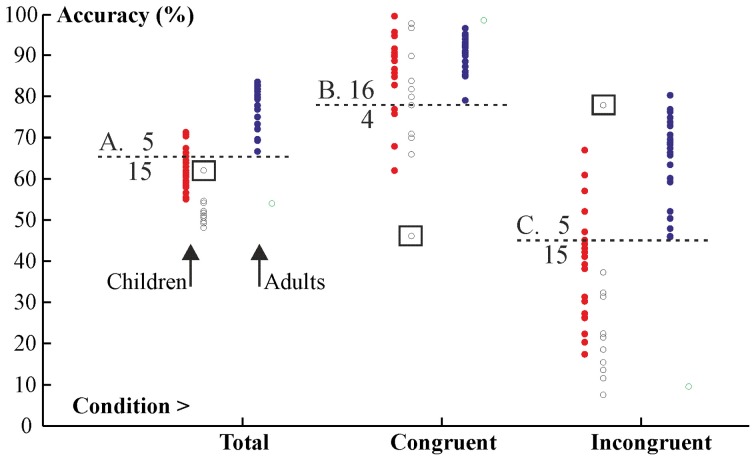
**Individual variability in performance.** Filled circles show participants included in analyses. Open circles show participants excluded from analyses. Children's data are on the left and adults' data are on the right. One excluded child participant with atypical data is marked by squares.

The importance of the Congruency factor is further demonstrated by examining individual variability in performance shown in Figure 5. This figure shows excluded participants as open circles and included participants as filled circles. It is well visible that the performance of excluded participants was at about the same level as that of included participants in the congruent condition. However, in general, their performance was substantially lower in the incongruent condition. Hence, most exclusions (due to weak overall performance) actually happened because of weak performance in the incongruent condition. That is, incongruent trials strongly influenced the data. This pattern also remained in the included participants. Overall, 15 out of 20 children (binomial test: *p* = 0.021) performed worse than the worst adult (mark “A” in Figure 5). However, only 4 children (*p* = 0.006) performed worse than the worse adult in the congruent condition (mark “B” in Figure 5) whereas 15 children performed worse than the worse adult in the incongruent condition (*p* = 0.021; mark “C” in Figure 5). Hence, it is clear that incongruent trials very strongly affected the data of included participants. It is important to note that only one excluded child participant showed an atypical pattern of having higher performance in the incongruent than in the congruent condition (marked by a square in Figure 5). This pattern probably arose due to misunderstanding instructions.

**Figure 6:**
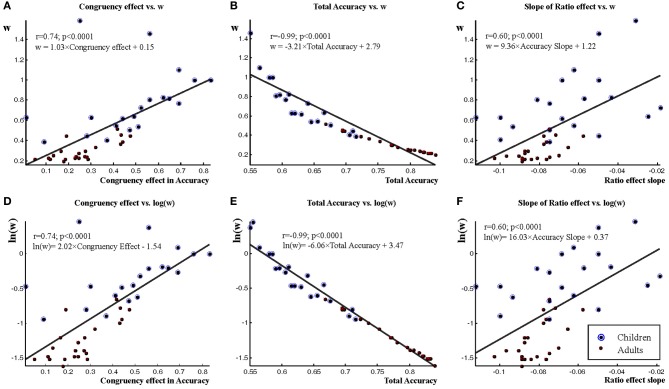
**The relationship of some measures and *w*.** The relationship of the congruency effect and *w* is shown in **A** and **D** (log transformed *w* data). The relationship of total accuracy and *w* is shown in **B** and **E** (log transformed *w* data). The relationship of the ratio effect slope and *w* is shown in **C** and **F** (log transformed *w* data). Correlations between the above measures and *w* are shown as well as equations for the regression lines represented in figures.

The relationship of the congruency effect and *w* is demonstrated in Figures 6A,D. In line with the above observations there was a strong correlation (*r* = 0.74; *p* < 0.001) between *w* (which is a direct function of accuracy) and the congruency effect. In other words, the congruency effect in a particular individual very strongly affected the *w*-value. The relationship of total accuracy and *w* is demonstrated in Figures 6B,E. There was a practically perfect correlation (*r* = −0.99; *p* < 0.001) between *w* and accuracy. On a logarithmic *w* scale the relationship was a near perfectly fitting linear function (Figure 6E). This is because *w* is an exponential based direct function of accuracy data. The relationship between the slope of the ratio effect and *w* is demonstrated in Figures 6C,F. There was a strong correlation between the two measures (*r* = 0.60; *p* < 0.001). This is unsurprising because the decision curves underlying *w* calculation also represent slope information.

**Figure 7:**
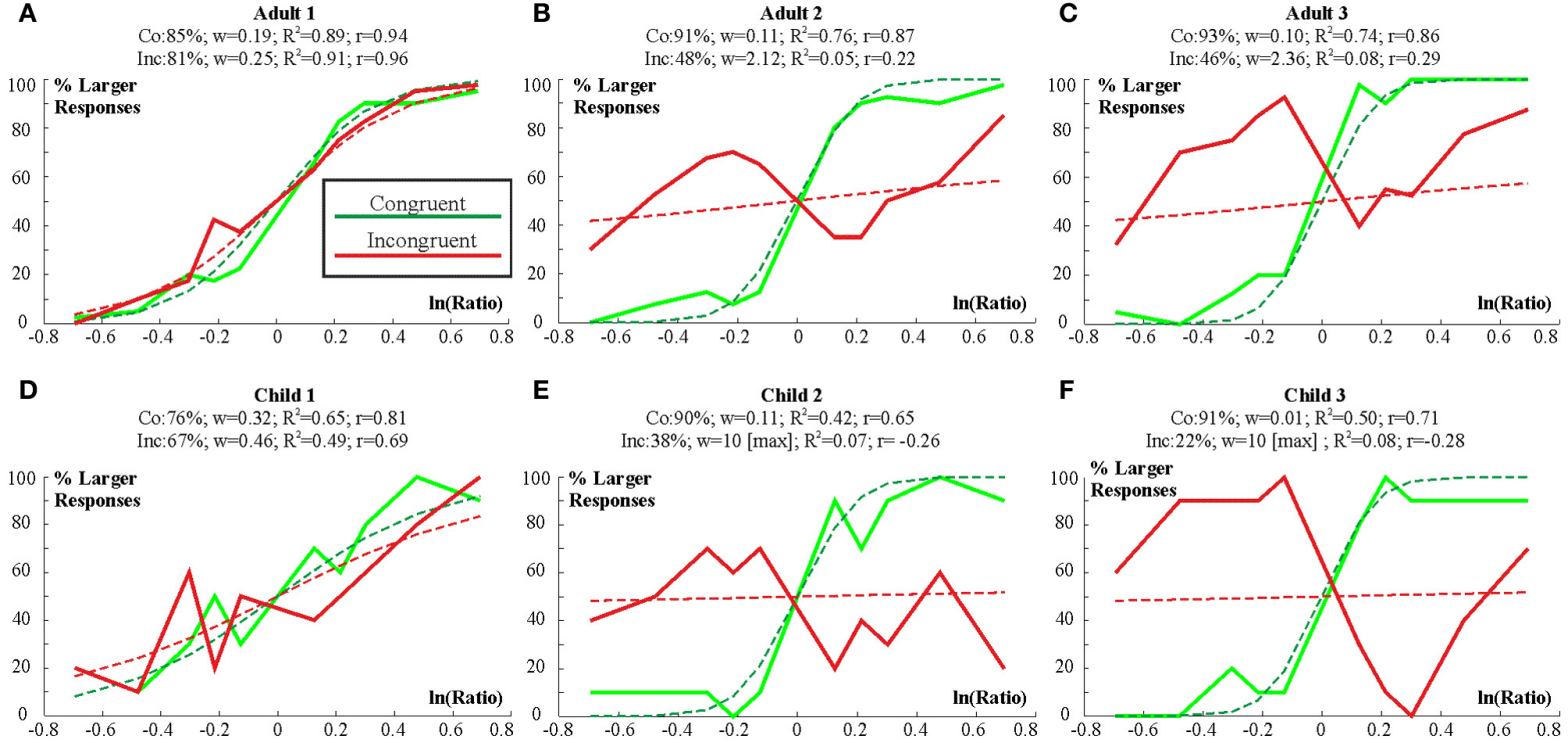
**Individual decision curves for 3 adult (A–C) and 3 child participants (D–F).** Empirical decision curves are shown by thick lines. Model decision curves are shown by thin dashed lines. Figure titles show the following information: Accuracy for the congruent (Co) and incongruent (In) conditions. *w* for both conditions. *R*^2^ (*R*^2^) for the *w* model fit and related correlation values (*r*).

Examples of representative individual participants are shown in Figure 7. Figure 7A shows an adult participant with excellent performance. Figure 7B shows an adult participant with excellent performance in the congruent condition but with chance performance in the incongruent condition. Figure 7C shows an adult participant with excellent performance in the congruent condition but with very low performance at ratios close to 1 which suggests that this participant tended to choose the visually “larger” stimulus at ratios close to 1. Figure 7D shows the best performing child participant. Figure 7E shows a child participant with excellent performance in the congruent condition but with chance performance in the incongruent condition. Figure 7C shows a child participant with excellent performance in the congruent condition but with very low performance at ratios close to 1. Again, this suggests that this child chose the visually “larger” stimulus at ratios close to 1. All participants fit into the above categories.

RT data was evaluated by a Group × Congruency × Ratio ANOVA. Results are illustrated in Figures 4C,D. Most ANOVA effects were significant. Children were 442 ms slower than adults [1104 ± 29 ms vs. 662 ± 27 ms; Group: *F*_(1, 40)_ = 124.1; η^2^_*p*_ = 0.76; *p* < 0.0001]. There was an effect of Congruency [*F*_(1, 40)_ = 108.5; η^2^_*p*_ = 0.73; *p* < 0.001] and a Congruency × Group interaction [*F*_(1, 40)_ = 14.6; η^2^_*p*_ = 0.27; *p* < 0.001]. All Congruency × Group Tukey *post-hoc* contrasts were significant (*p* < 0.001; Children congruent vs. incongruent: 1011 ± 59 vs. 1198 ± 75 ms; adults: 619 ± 56 vs. 705 ± 71 ms). There was a Ratio effect [*F*_(4, 160)_ = 3.5; ε = 0.854; η^2^_*p*_ = 0.86; *p* < 0.001] and there were, Congruency × Ratio [*F*_(4, 160)_ = 3.8; ε = 0.678; η^2^_*p*_ = 0.08; *p* = 0.014] and Group × Congruency × Ratio [*F*_(4, 160)_ = 4.94; ε = 0.678; η^2^_*p*_ = 0.11; *p* = 0.004] interactions (Group × Ratio: *p* = 0.62).

## Discussion

It has been proposed that the acuity of the ANS can be accurately characterized by a parameter, *w*, computed from psycho-physical decision curves in non-symbolic magnitude discrimination tasks. In order to validate that above claim, we investigated the impact of congruency between numerical and visual stimulus parameters on *w*. We found that number discrimination performance and *w* are strongly influenced by visual stimulus parameters. Consequently, visual parameter confounds in ANS tasks can substantially distort developmental data when comparing children to adults and when comparing different child groups.

In the non-symbolic number discrimination task we manipulated/controlled the stimuli in two different manners: across trials participants could not rely on a single visual stimulus property to judge numerosity and the difference in visual stimulus properties and numerosity of the two dot arrays presented concurrently did not correlate across trials. Furthermore, we controlled for more visual stimulus parameters than related studies. Visual stimulus properties had strong impact on performance. Both adults and children performed equally well in the congruent condition even in the most difficult ratio conditions. However, performance dropped significantly for the incongruent condition and was below chance (40%) for the hardest ratio (0.88) even in adults and at all ratios except at the easiest one (0.5) in children (20–45%). At ratios 0.74, 0.81, and 0.88, children followed visual parameters in their “larger” choice in 70–80% of trials.

In the incongruent condition of our task one response option was the numerically larger but with regards to visual cues “less” magnitude. The other response option was the less numerous but with visually “more” magnitude. That is, completely random performance would have been characterized by 50% accuracy. Had participants chosen the more numerous display it would have manifested itself in >50% accuracy. In contrast, <50% accuracy indicates that participants mostly chose the “wrong” response option with visually “more” magnitude. Hence, the low accuracy in the incongruent condition in our data suggests that when visual parameters are controlled more than usual, even adults find it hard to extract numerical information from a display and some 7-year-old children find it impossible at all but the easiest 0.5 ratio to discriminate numerosities of dot patterns. Findings are in line with some other studies investigating the effect of congruency on non-symbolic numerosity comparison (Rousselle et al., 2004; Soltész et al., 2010).

It is important to point out that the below chance performance in the incongruent condition is not a problem of our experimental stimulus design. Our experimental task was particularly difficult because of the very stringent control of visual stimulus properties, which are necessary if we want to assess how well people are able to determine number when visual cues are controlled. In other words, it was exactly one of our empirical questions whether under more stringent visual controls children and adults are still influenced by the sensory cues present in the stimuli. Moreover, it is unavoidable that there will be trials with congruent and incongruent numerical and visual information when trying to control the visual stimulus parameters in a numerical discrimination task. Therefore, congruency effects are present in all studies applying some sort of control on the sensory cues of numerosity stimuli, but no thorough research has been done to examine their influence on performance. Importantly, it is impossible to get rid of confounding visual parameters in particular individual trials. In order to get an indication of their influence on performance, it is therefore necessary to investigate congruency effects in an explicit manner. All experiments lacking such an analysis cannot provide reliable information about numerosity processes.

The more stringent control of visual cues in our experiment than previously used resulted in increased task difficulty as shown by the larger *w*-values in the current than previous studies. Piazza et al. (2010) reported *w*-values of 0.15 for adults; 0.25 for 10-year-old children and 0.34 for children with dyscalculia. Mazzocco et al. (2011) determined *w*-values of about 0.28 for 14/15 year-old children and 0.36 for children with dyscalculia. Here, for the overall results (congruent and incongruent trials together) we measured *w*-values of 0.3 for adults and 0.77 for 7-year-old children. The fact alone that performance and therefore *w* are dependent on the method used to control the visual stimulus properties suggests that numerosity estimation processes are not independent of visual stimulus properties. In other words, *w*-values cannot be considered to reflect the acuity of pure numerosity processes. In fact, under the visual control circumstances we used here, the model underlying *w* computation provided a generally poor fit to the data in the incongruent condition where decision curves frequently fall below chance in individual participants even in adults (reflecting the impact of visual confounds on number discrimination).

Another important conclusion regards the observation that performance in the incongruent condition was worse in children than in adults (Gebuis et al., 2009; Soltész et al., 2010). This implies that adults can better cope with the inconsistent visual stimulus information than children (see for a similar view: Defever et al., 2013). Because *w* is computed as a direct function of accuracy and in fact shows near perfect correlation with total accuracy (see Figure 6) it is not surprising that the *w*-values for incongruent trials were much larger for children (7.10) than adults (0.74). Furthermore, the *w* for congruent trials did not differ significantly in children (0.18) and adults (0.12). From the above results it logically follows that *w* computed for the overall data (congruent and incongruent) was worse for children than adults because of the impact of the incongruent condition on *w*. In fact, *w* was strongly correlated (*r* = 0.74) with the congruency effect derived from the accuracy data. The larger was the congruency effect, the larger was *w*. Thus, *w* is strongly influenced by congruency, and is in fact a fairly clear function of congruency. Consequently, studies that do not explicitly examine the effect of visual parameters might have a serious confound in their results. Namely, there is a strong possibility that *w* and accuracy differences observed between children and adults are partly or mainly due to differences in congruency effects between adults and children. If children show a larger congruency effect than adults, total accuracy will drop and *w* will increase more in children relative to adults. While congruency effects were much larger in our experiment than in related experiments (because of more stringent visual parameter controls), it does not mean that congruency effects would become negligible in other experiments. As long as congruency effects result in significant accuracy differences between congruent/incongruent conditions (e.g., Halberda and Feigenson, 2008), it is highly likely that this influence of the sensory cues will significantly impact on *w* as well. Importantly, it has been demonstrated that manipulating different visual stimulus properties in opposite directions can cancel out the congruency effect. This, however, does not imply that the visual stimulus properties did not affect performance. In this case, the influence of the visual stimulus properties is only masked by the opposite effects induced by the different visual stimulus properties (Gebuis and Reynvoet, 2012a).

There was substantial individual variability in our data. First, it is notable that when using the criterion level of Piazza et al. (2010), i.e., *R*^2^ < 0.2, we had to reject about 30% of our sample of 7-year-old children. In effect, this relatively arbitrary criterion level means that total accuracy was ≤54%. Two further children and one adult were also excluded because of ≤55% total accuracy level. Overall, it is striking that more than 30% of 7-year-old children who already have firm knowledge of symbolic number (Rubinsten et al., 2002) and finished the first year of primary school, as well as one adult with average mathematical competence (with a university degree) were unable to solve the task appropriately. The analysis of their errors demonstrated that their overall weak performance was due to very low accuracy in the incongruent condition (8–40%; see Figure 5). Similarly low accuracy was reported in less controlled paradigms, e.g., in an easier task Piazza et al. (2010) reported excluding 18 out of 44 (41%) kindergarten children for low response accuracy. The high level of exclusions does not seem compatible with an ANS functioning in an obligatory manner from an early age. Rather, the data seem to suggest that people are primarily responding to sensory cues and cannot extract numerosity independent of the sensory cues. In addition, our data suggest that most exclusions are related to poor performance in incongruent trials. That is, the practice of excluding large numbers of young children from data analysis without examining why they were excluded (like we did here) artificially boosts confidence in ANS related explanations. This is because the practice completely neglects that children were probably excluded because they could not ignore visual cues (and hence, they did not fit the ANS model). Considering that e.g., nearly half the kindergarten children were excluded in Piazza et al. (2010) this seems a significant problem rather than affecting only a small portion of child data. The high proportion of excluded young children also seems incompatible with the view of an early functioning ANS.

With regard to variability it is important to realize that here we used a particularly powerful visual stimulus manipulation. This resulted in extremely poor performance in the incongruent condition. However, the much better accuracy in the congruent condition was still enough to raise overall model fit above *R*^2^ = 0.2 and total accuracy above 55%. Hence, it is clear that in experiments with no explicit congruent/incongruent conditions there is a high chance for the visual stimulus parameters to strongly affect the data without having a major detrimental effect on total accuracy. That is, several participants with strong congruency effects may be included in the final sample of participants. Another comment regards performance in the congruent condition. This condition does not necessarily reflect true number discrimination performance. Similar to the incongruent condition, the congruent condition reflects performance in a number discrimination task confounded by visual cues.

It could be argued that perhaps our study had unusual stimulus presentation parameters, which may make comparison with ANS studies hard. However, studies aiming to measure ANS acuity used various presentation parameters and nevertheless reported similar data. For instance, some presented two dot patterns sequentially following each other in time (Piazza et al., 2004). Others presented stimuli simultaneously with two differently colored dot patterns intermixed (Halberda et al., 2008; Mazzocco et al., 2011), while others presented stimuli simultaneously with the two dot patterns on the left and the right side of the fixation (Halberda and Feigenson, 2008; Piazza et al., 2010). Some used brief presentation times of 150 ms (Piazza et al., 2004) and 200 ms (Halberda et al., 2008; Mazzocco et al., 2011) while others used long presentation times. Piazza et al. (2010) left stimuli on the screen until participants gave a response and Halberda and Feigenson (2008) presented each stimulus for a duration of 2000 Ms. Overall, the nature of presentation (simultaneous or sequential; side by side or intermixed) and presentation time (150, 200, 2000 ms and until response) does not seem to affect *w* data. In fact, recently Price et al. (2012) reported similar results and reliability for *w* in intermixed, side-by-side (paired) and sequential trial presentations formats in adults. Moreover, in our experiment we presented stimuli for 2000 ms with the to-be-compared dot patterns side-by-side, that is, in the same way as Halberda and Feigenson (2008). Hence, it seems that our results are generalizable to non-symbolic magnitude discrimination tasks.

As described in the Introduction, a magnitude discrimination task with conflicting stimulus dimensions is practically a Stroop task. Congruency effects can appear because participants do rely on visual properties rather than on number *and* because they may find it hard to resolve (implicitly or explicitly) the conflict between numerical and non-numerical (visual) stimulus dimensions and/or they cannot inhibit irrelevant response tendencies efficiently. Clearly, the large congruency effect in our data cannot be explained completely by response inhibition effects. Namely, in an animal size decision Stroop task where physical size is a very strong and “natural” distracter over real life size we found congruency (congruent vs. incongruent) effects in accuracy with an effect size of 11% in 5-year-olds and about 3% in 8-year-olds and adults (Bryce et al., 2011). In contrast, in the current experiment the congruency effect was 47.5% in children and 26% in adults. Hence, it is likely that the major part of congruency effects in this study was due to relying on visual cues instead of number when making supposedly number-specific decisions. Further, it is unlikely that the response inhibition difference between adults and children can fully explain the group difference and most possibly children relied much more than adults on visual stimulus properties to judge numerosity. This is also supported by the reaction time data, which does not reflect the usual numerical ratio effect in the incongruent condition of the children.

While children in our study genuinely relied on visual cues instead of number in many trials, it is also well known that children have worse inhibitory function than adults (Gerstadt et al., 1994; Bunge et al., 2002; Prevor and Diamond, 2005; Szűcs et al., 2007, 2009; Bryce et al., 2011). Hence, inhibitory control differences between children and adults can also affect results in ANS tasks and may explain part of the findings here. Inhibitory control develops throughout childhood and especially from kindergarten to school age (Gerstadt et al., 1994; Prevor and Diamond, 2005). Hence, it is very likely that developmental ANS studies not explicating congruency effects in young children confound putative ANS related effects with developmental changes in congruency effects due to the development of inhibitory function. In relation to this, a recent study demonstrated that ANS acuity explained a small but significant amount of variance in mathematics in children but only when trials from an incongruent condition were taken into account (Fuhs and McNeil, 2013). In fact, the relationship between ANS acuity and mathematical performance ceased to be significant when inhibitory control ability was also taken into account. This finding is in perfect agreement with our data and suggests that even when an ANS task is easier than the one used here, inhibitory demands in the incongruent condition have a major impact on the relation of the ANS task and mathematical performance. In addition, it is also well documented that inhibitory control plays an important role in mathematical performance (McKenzie et al., 2003; Espy et al., 2004; Blair and Razza, 2007; Bull and Scerif, 2001; Swanson, 2011) and that children with developmental dyscalculia show impaired inhibitory control (Bull et al., 1999; Passolunghi et al., 1999; Passolunghi and Siegel, 2004). Hence, when ANS studies compare child populations with and without developmental dyscalculia they may measure inhibitory control differences between child populations rather than differences in ANS acuity. In fact, Szűcs et al. (2013) demonstrated increased congruency effects in children with developmental dyscalculia in an ANS task while measuring generally worse inhibitory control in children with dyscalculia than in controls. This explicitly suggests that impaired inhibitory control in dyscalculia can affect performance in ANS tasks.

Overall, we conclude that accuracy in non-symbolic number comparison, and thus *w*, is seriously influenced by the visual display parameter confounds (congruency between visual and numerical parameters). Further research is necessary to decide to what extent non-symbolic comparison may rely on an ANS or rather, non-symbolic comparison is fully determined by the analysis of visual cues, or by a mixture of potential ANS processes and visual feature analysis. We demonstrate that visual parameter confounds in ANS tasks can substantially distort developmental data when comparing children to adults and when comparing different child groups. That is, previous studies which did not explicitly examine the impact of visual parameters on *w* and interpreted their results in terms of true ANS-related effects may have reached inadequate conclusions. This is because differences in *w* may have been the consequence of differential sensitivity of adults and children to visual display parameters (differences in congruency effects) and/or the differential developmental level of inhibitory control in child and adult populations. Hence, we suggest that the conclusions and data from such studies may not, or only partially, reflect developmental effects related to the ANS and should be interpreted with caution.

### Conflict of interest statement

The authors declare that the research was conducted in the absence of any commercial or financial relationships that could be construed as a potential conflict of interest.
